# Let's retire phenytoin! Levetiracetam as the first choice in the treatment of convulsive status epilepticus

**DOI:** 10.1055/s-0046-1817042

**Published:** 2026-03-12

**Authors:** Luis Otávio Caboclo, Carol Ladeia Lopes Frota

**Affiliations:** 1 Hospital Israelita Albert Einstein, São Paulo SP, Brazil.

**Keywords:** Status Epilepticus, Levetiracetam, Phenytoin, Adverse Effects

## Abstract

Status epilepticus (SE) is the most severe presentation of epilepsy. Most available data on the treatment of SE refers to the convulsive type. Treatment begins with an accurate diagnosis, followed by clinical stabilization and rapid administration of benzodiazepines (BZDs). Then, treatment continues with the use of an intravenous antiseizure medication (ASM). Phenytoin has been the preferred ASM as the second-line treatment of SE (after failure of BZDs to control seizures), although this choice was mostly guided by low-quality evidence. Recent evidence demonstrates that levetiracetam is at least as effective as phenytoin in the treatment of convulsive SE, both in adults and children. Moreover, growing evidence indicates that levetiracetam offers better tolerability and a more favorable safety profile. Therefore, in this Point of View, we argue that we should retire phenytoin in the treatment of convulsive SE, and levetiracetam should become the first choice of treatment.

Status epilepticus (SE) is the most severe presentation of epilepsy. It affects all age groups, though with different etiologies and outcomes, and it carries higher mortality and morbidity in elderly people, while it has better outcomes in children and adolescents. Currently, SE is defined as “a condition resulting either from the failure of the mechanisms responsible for seizure termination or from the initiation of mechanisms which lead to abnormally-prolonged seizures (after time point T1).” 1 Moreover, “it is a condition that can have long-term consequences (after time point T2), including neuronal death, neuronal injury, and alteration of neuronal networks, depending on the type and duration of seizures”. 1 Therefore, two points of time are defined in the evolution of prolonged seizures. For the most severe type of SE—convulsive SE—these time points are better defined, and have clinical significance: T1 is 5 minutes, since after this time it is unlikely that the seizure will resolve spontaneously, therefore requiring initiation of therapy; and T2 is 30 minutes, since seizures that last beyond this time are more likely to cause long-term consequences. From a clinical point of view, treatment should be started at T1, so that the seizure does not last until T2. For other types of seizures, evidence supporting these time frames is less conclusive.

Status epilepticus should be diagnosed and treated promptly, as early treatment is associated with improved outcomes. 2 3 Most available data on the treatment of SE pertains to the convulsive type, whereas evidence regarding the management of nonconvulsive SE remains considerably more limited. The treatment of convulsive SE begins with an accurate diagnosis, followed by clinical stabilization and the administration of antiseizure medication (ASM), aiming at stopping the seizure and preventing secondary neurological damage.

The first phase of treatment of convulsive SE is the rapid administration of benzodiazepines (BZDs). 4 The choice of BZD must be based on existing guidelines and availability. Intravenous (IV) lorazepam, IV diazepam, or intramuscular (IM) midazolam are recommended. For patients without established IV access, IM midazolam is preferred to avoid delays in initiating treatment. 4 5

The initiation of a second-line ASM is indicated if the seizures persist after one or two adequately-dosed BZD administrations. Moreover, even if the seizures respond to BZD, second-line medications should also be administered to prevent seizure recurrence, 6 as treatment with BZD alone carries a high risk of recurrence, 5 7 maybe with the exception of patients in whom a rapidly-reversible cause of SE has been identified and treated, such as severe hyponatremia or hypoglycemia. 6

During this phase, an IV loading dose of an ASM should be administered. The IV options include phenytoin (PHT) or its prodrug fosphenytoin (fPHT), levetiracetam (LEV), valproate (VPA), phenobarbital (PB) and lacosamide (LCM).

Since the introduction of its parenteral formulation in 1950, PHT has been the predominant ASM employed as second-line treatment for convulsive SE. 8 However, the choice of PHT over another ASM has been mostly guided by low-quality evidence. 9 10 More recent studies, however, have challenged the notion that PHT should be preferred over other second-line ASMs.

In 2014 a meta-analysis 9 evaluated the published evidence-base of the efficacy of the five IV ASMs in BZD-resistant convulsive SE. The authors concluded that the use of PHT in this setting was not supported by evidence; LEV, VPA and PB were recommended as preferred agents. 9 Intravenous ASMs were also compared in a meta-analysis 10 assessing their cost and effectiveness in BZD-resistant convulsive SE; in this analysis, PHT and fPHT were the least effective agents, with a probability of stopping the seizures of 0.53.

In 2019, the Established Status Epilepticus Treatment Trial (ESETT) provided high-quality evidence for the choice of second-line treatment of SE. 11 In this trial, 384 pediatric and adult patients with convulsive SE refractory to BZDs were enrolled and randomly allocated to receive either fPHT (20 mg/kg, phenytoin equivalents), VPA (40 mg/kg), or LEV (60 mg/kg, all loaded intravenously). The outcome of the study was cessation of SE and improvement in the level of consciousness at 60 minutes, which was achieved in 47% of the LEV group, 46% of the VPA group, and 45% of the fPHT group. In conclusion, the three ASMs were equally effective and presented similar rates of adverse effects. This study supported the use of LEV as an alternative to PHT/fPHT for the treatment of BZD-resistant convulsive SE, particularly in settings in which IV VPA is unavailable. 12

Several subsequent studies have compared LEV and PHT as second-line treatments for SE. Following the ESETT, two studies compared the effectiveness of PHT and LEV in the treatment of convulsive SE refractory to BZD in children. The Levetiracetam versus phenytoin for second-line treatment of convulsive status epilepticus in children (ConSEPT) trial 13 was an open, multicenter, randomized trial conducted in centers in Australia and New Zealand. The Emergency Treatment with Levetiracetam or Phenytoin in Status Epilepticus in Children (EcLiPsE) trial, 14 also an open, multicenter, randomized trial, was conducted in 30 centers in the United Kingdom. In both trials, children aged from 3 months to 16 years (ConSEPT) and from 6 months to 18 years (EcLiPsE) were randomly assigned to receive LEV (40 mg/kg) or PHT (20 mg/kg). In the ConSEPT trial, the primary outcome was clinical cessation of seizure activity 5 minutes after the completion of infusion of the study group, which was achieved in 68 (60%) patients in the PHT group and in 60 (50%) patients in the LEV group ( *p* = 0.16, not statistically significant). In the EcLiPsE trial, the primary outcome was time to cessation of convulsive SE, which was terminated in 106 (70%) children in the LEV group and in 86 (64%) in the PHT group. The median time from randomization to cessation of convulsive status epilepticus was of 35 minutes in the LEV group and 45 minutes in the PHT group ( *p* = 0.20, not statistically significant). In both trials, LEV was equivalent to PHT in terms of seizure cessation and adverse event rates, although LEV demonstrated a trend toward a more favorable safety profile and ease of administration, making it an appropriate alternative to PHT as the first-choice second-line ASM in the treatment of pediatric convulsive SE.

With respect to adverse effects, recent data has shown that LEV may be preferred to PHT. A meta-analysis 15 compared LEV and PHT in the treatment of SE and found that LEV was more effective and associated with a lower incidence of major adverse effects. Two recent systematic reviews and meta-analyses 16 17 confirmed this data in pediatric SE. In a meta-analysis including 14 studies, 16 LEV was compared with other drugs used in SE treatment in children (PHT/fPHT and VPA); LEV was associated with reduced time to cessation of seizures (compared with PHT/fPHT) and shorter length of intensive care unit stay (compared with PHT). Moreover, a lower risk of adverse events was noted with LEV compared with PHT. 16 Another meta-analysis, 17 comparing the efficacy and safety of LEV versus PHT/fPHT as second-line treatments in children with convulsive SE, found that LEV was as effective as PHT/fPHT to control seizures, and it was associated with fewer seizure recurrences and adverse events.

Safe and easy to use, the loading dose of LEV may be infused in 5 to 15 minutes. 18 19 The rapid infusion (5 minutes) of doses up to 4,500 mg IV is safe and well tolerated. 18 20 Due to its safety profile and pharmacological characteristics, LEV may be preferred in patients with liver failure, cardiac arrhythmias (which can be aggravated by IV PHT) or hemodynamic instability. 21 22 The absence of significant drug interactions and a favorable tolerability profile are additional advantages when transitioning to long-term maintenance therapy. Table 1 summarizes the intravenous LEV dosing regimens for pediatric and adult patients, as reported in the aforementioned studies 11 13 14 comparing LEV with other antiseizure medications.

**Table 1:** Levetiracetam dosing in children and adults with convulsive status epilepticus

Trial	Loading dose	Maximum dose	Age	Route	Infusion rate
ESETT 11	60 mg/kg	4,500 mg	1–94 years	IV	10 minutes
ConSEPT 13	40 mg/kg	3,000 mg	3 months–16 years	IV or IO	5 minutes
EcLiPsE 14	40 mg/kg	3,000 mg	6 months–18 years	IV or IO	5 minutes

Abbreviations: ConSEPT, levetiracetam versus phenytoin for second-line treatment of convulsive status epilepticus in children; EcLiPSE, Emergency Treatment with Levetiracetam or Phenytoin in Status Epilepticus in Children; ESETT, Established Status Epilepticus Treatment Trial; f-PHT, fosphenytoin; IO, intraosseous; IV, intravenous; PHT, phenytoin; VPA, valproic acid.

In contrast to LEV, PHT has a narrow therapeutic window and a wide range of dose-related and idiosyncratic adverse effects, including common and serious reactions. 23 The most common adverse effects of PHT involve the nervous system and are often dose-related. Acute manifestations include nystagmus, ataxia, dysarthria, impaired coordination, somnolence, and cognitive slowing. Other neurologic symptoms may include dizziness, vertigo, insomnia, paresthesias, and headaches. 24 Dermatologic reactions are also a great concern, and they may vary from mild rashes to severe, potentially-fatal conditions such as Stevens-Johnson syndrome, toxic epidermal necrolysis, and drug reaction with eosinophilia and systemic symptoms (DRESS). 23 25

Accumulating evidence demonstrates that LEV is at least as effective as PHT for BZD-resistant convulsive SE, with recent studies favoring LEV due to its superior tolerability and safety profile. Why, then, should PHT continue to be used in this clinical setting? The answer is: it should not. Cost has been cited as a potential drawback of LEV. Nonetheless, although data remain limited, available evidence suggests that LEV may actually be more cost-effective than PHT in the management of status epilepticus. 10 In conclusion, it is time to retire PHT in the treatment of convulsive SE. There is now sufficient evidence to recommend LEV as the first-line treatment for BZD-resistant convulsive SE. Phenytoin should now be considered among the historical treatments that, although once pivotal, ought to be replaced by more effective, safer, and more cost-effective alternatives.
